# Differences in biopsychosocial profiles of diabetes patients by level of glycaemic control and health-related quality of life: The Maastricht Study

**DOI:** 10.1371/journal.pone.0182053

**Published:** 2017-07-27

**Authors:** Arianne M. J. Elissen, Dorijn F. L. Hertroijs, Nicolaas C. Schaper, Hans Bosma, Pieter C. Dagnelie, Ronald M. Henry, Carla J. van der Kallen, Annemarie Koster, Miranda T. Schram, Coen D. A. Stehouwer, Johannes S. A. G. Schouten, Tos T. J. M. Berendschot, Dirk Ruwaard

**Affiliations:** 1 Department of Health Services Research, Maastricht University, Maastricht, The Netherlands; 2 CAPHRI Care and Public Health Research Institute, Maastricht University, Maastricht, The Netherlands; 3 Department of Internal Medicine, Maastricht University Medical Centre+, Maastricht, The Netherlands; 4 CARIM School for Cardiovascular Diseases, Maastricht University, Maastricht, The Netherlands; 5 Department of Social Medicine, Maastricht University, Maastricht, The Netherlands; 6 Department of Epidemiology, Maastricht University, Maastricht, The Netherlands; 7 Heart and Vascular Centre, Maastricht University Medical Centre +, Maastricht, The Netherlands; 8 University Eye Clinic Maastricht, Maastricht University Medical Centre+, Maastricht, The Netherlands; Tecnologico de Monterrey, MEXICO

## Abstract

**Aims:**

Tailored, patient-centred innovations are needed in the care for persons with type 2 diabetes mellitus (T2DM), in particular those with insufficient glycaemic control. Therefore, this study sought to assess their biopsychosocial characteristics and explore whether distinct biopsychosocial profiles exist within this subpopulation, which differ in health-related quality of life (HRQoL).

**Methods:**

Cross-sectional study based on data from The Maastricht Study, a population-based cohort study focused on the aetiology, pathophysiology, complications, and comorbidities of T2DM. We analysed associations and clustering of glycaemic control and HRQoL with 38 independent variables (i.e. biopsychosocial characteristics) in different subgroups and using descriptive analyses, latent class analysis (LCA), and logistic regressions.

**Results:**

Included were 840 persons with T2DM, mostly men (68.6%) and with a mean age of 62.6 (±7.7) years. Mean HbA1c was 7.1% (±3.2%); 308 patients (36.7%) had insufficient glycaemic control (HbA1c>7.0% [53 mmol/mol]). Compared to those with sufficient control, these patients had a significantly worse-off status on multiple biopsychosocial factors, including self-efficacy, income, education and several health-related characteristics. Two ‘latent classes’ were identified in the insufficient glycaemic control subgroup: with low respectively high HRQoL. Of the two, the low HRQoL class comprised about one-fourth of patients and had a significantly worse biopsychosocial profile.

**Conclusions:**

Insufficient glycaemic control, particularly in combination with low HRQoL, is associated with a generally worse biopsychosocial profile. Further research is needed into the complex and multidimensional causal pathways explored in this study, so as to increase our understanding of the heterogeneous care needs and preferences of persons with T2DM, and translate this knowledge into tailored care and support arrangements.

## Introduction

Diabetes care in the Netherlands is widely regarded as a ‘best practice’ [1] and several developments were pivotal in shaping this care model. In 2003, an evidence-based standard for generic care for type 2 diabetes mellitus (T2DM) was established by the Netherlands Diabetes Federation–an umbrella organisation of diabetes care professionals, patients and researchers–providing the norm for high-quality, multidisciplinary diabetes care [2].

Another important change followed in 2007, when a bundled payment system was introduced allowing health insurers to contract the different components of generic diabetes management as an integrated care programme, based on the diabetes care standard [3–5]. Their main contracting partners in primary care are care groups, i.e. networks of general practitioners (GPs) comparable to Clinical Commissioning Groups (CCGs) in the United Kingdom. As part of their contract with health insurers, care groups assume clinical and financial responsibility for integrated diabetes care delivery and coordination [6]. Today, there are around 115 care groups with an integrated diabetes care contract, covering 85 percent of the approximately 900,000 Dutch citizens with diagnosed T2DM [6,7].

Since care groups emerged in Dutch primary care, many studies have been conducted to assess the quality of diabetes care provided by these groups. According to a recent evaluation [6], relevant process and outcome indicators have improved over the years in most groups and now seem to be stabilising. For example, a relatively steady share of around two-thirds of patients has sufficient glycaemic control (glycated haemoglobin (HbA1c) levels ≤7.0% [53 mmol/mol]) [6]. Within the limitations of current practice, it seems unlikely that this percentage will increase much further: both the former report [6] and the Euro Diabetes Index [1] showed that in general, Dutch GPs strictly adhere to the care standard, suggesting that the outcomes achieved represent near-optimal results.

The existence of plateau values in processes and outcomes points towards a need for further innovation: the current, highly standardised care approach leaves a considerable subgroup–about a third of patients with diagnosed T2DM, i.e. roughly 300,000 people in the Netherlands [6,7]–unable to adequately manage glycaemic control. In the long-term, these patients have a higher risk of microvascular and macrovascular complications, and lower health-related quality of life (HRQoL) [8]. The phenomenon of differential treatment effects is not unique to Dutch diabetes care: multiple studies in different countries have recently shown that ‘one-size-fits-all’ diabetes management does not actually fit for all patients [9,10]. It remains unclear, however, which biopsychosocial factors are associated with more or less promising treatment outcomes.

The present study hypothesises that there is a broad range of patient characteristics influencing the ability of individuals to self-manage, their need for professional treatment and support, and, ultimately, their level of glycaemic control and HRQoL. In a first step towards leveraging these characteristics to develop more person-centred, tailored diabetes care, this study aims to: (1) gain insight into the biopsychosocial characteristics of patients with insufficient glycaemic control, as opposed to patients with sufficient control; and (2) explore whether distinct biopsychosocial profiles can be identified within the group of patients with insufficient glycaemic control, which are associated with different HRQoL. For the latter purpose, an explorative latent class analysis (LCA) was conducted. The study was based on a comprehensive subset of phenotyping data from the population-based The Maastricht Study.

## Materials and methods

### Study design and study population

We conducted a cross-sectional study based on data from The Maastricht Study, an observational prospective population-based cohort study in the region of Maastricht in the southern part of the Netherlands. The rationale and methodology have been described previously [11]. In brief, the study focuses on the aetiology, pathophysiology, complications, and comorbidities of T2DM, and is characterised by an extensive phenotyping approach. Eligible for participation were all individuals aged between 40 and 75 years, and living in the Maastricht region. Participants were recruited through mass media campaigns and from the municipal registries and the regional Diabetes Patient Registry via mailings. Recruitment was stratified according to known T2DM status, with an oversampling of individuals with T2DM, for reasons of efficiency.

For this study, cross-sectional data were used from the first 975 participants with T2DM in The Maastricht Study, who completed the baseline survey between November 2010 and September 2013. The examinations of each participant were performed within a time window of three months. Participants were included in the present study if they were previously diagnosed with T2DM by a health professional (i.e. prior to participating in The Maastricht Study) and had an HbA1c measurement conducted at The Maastricht Study research centre. No further in- or exclusion criteria were used.

The Maastricht Study has been approved by the institutional medical ethical committee (NL31329.068.10) and the Minister of Health, Welfare and Sports of the Netherlands (Permit 131088-105234-PG). All participants gave written informed consent.

### Definition of dependent and independent variables

The study was conducted in two steps, which differed in terms of the dependent variable. First, to gain insight into differences in patients’ biopsychosocial characteristics by level of glycaemic control, we used participants’ HbA1c level as dependent variable. Although there is growing interest in, amongst others, glycated albumin and fructosamin as alternative markers of glycaemic control, HbA1c remains the gold standard biomarker of glycaemia [12]. It has been used as a universally accepted means for monitoring glycaemic control for more than three decades [13].

We dichotomised HbA1c based on the norm values in the Dutch diabetes care standard [2]. Thus, subgroups represented sufficient glycaemic control (HbA1c≤7.0% [53 mmol/mol]) versus insufficient glycaemic control (HbA1c>7.0% [53 mmol/mol]).Second, we explored whether there are distinct biopsychosocial profiles within the patient subgroup with insufficient glycaemic control, which differ in terms of HRQoL. Several HRQoL measures were used as dependent variable, given the potential effect of insufficient glycaemic control on HRQoL and the importance of this outcome to patients [8]. As LCA requires a categorical dependent variable, we dichotomised summary scores from three surveys focused on various domains of HRQoL: PAID, EQ-5D-3L and SF-36. The 20-items PAID (Problem Areas in Diabetes) survey assesses diabetes-related emotional distress; a sum score of 40 –indicating severe distress at the level of ‘emotional burnout’–was used for dichotomisation [14]. Based on the EQ-5D-3L questionnaire, five binary variables were defined illustrating the presence or absence of problems related to mobility, self-care, usual activities, pain/discomfort and anxiety/depression [15]. Participants’ SF-36 scores were aggregated into two summary measures of HRQoL, i.e. the Physical (PCS) and Mental Component Summary (MCS) scores [16]. The Dutch PCS and MCS norm scores–i.e. 50 and 42 points, respectively–were used as cut-off points for dichotomisation [17].

In both steps, independent variables comprised a comprehensive set of biopsychosocial characteristics considered potential predictors of health outcomes (in this case, glycaemic control and HRQoL) in patients with T2DM. To structure these characteristics in a meaningful way, we used Andersen and Newman’s Behavioural Model of Health Service Use [18]. Given the strong reported associations between glycaemic control, HRQoL and health service use [19,20], we assumed that applying this model could provide relevant insights for tailoring diabetes care. Anderson and Newman [18] distinguish three categories of individual determinants of health service use: person-related, context-related and health-related factors.

#### Person-related characteristics

Person-related (or predisposing) characteristics determine people’s personal predisposition to use health services [18]. The variables in this category were: age (in years), sex (male/female), smoking behaviour, alcohol consumption, self-reported physical activity (in hours/week), mastery, self-efficacy and social adequacy. Smoking behaviour was categorised as non-, former or current smoker. Alcohol consumption was classified as none, low (≤7 glasses/week for women; ≤14 glasses per week for men) or high (>7 glasses/week for women; >14 glasses per week for men) based on the 2006 Health Council of the Netherlands guidelines for a healthy diet [21]. Self-efficacy and mastery are measures of a person’s control beliefs: where self-efficacy is a person’s belief that he is able to perform a (desired) action or behaviour, mastery refers to his belief that his actions matter for outcomes.[22] We measured self-efficacy by the sum of items scores on the Dutch adaptation [23] of the validated, 16-item Self-Efficacy Scale of Sherer et al. [24]: higher scores suggest more self-efficacy. Mastery was defined as participants’ sum score on seven items of the Pearlin Mastery Scale, with higher total scores indicating a greater sense of personal mastery [25]. Social adequacy was measured using a shortened version (15 items) of the Dutch Personality Questionnaire, which was recoded so that higher sum scores indicate greater social adequacy [26].

#### Context-related characteristics

Context-related (or enabling) factors are largely socioeconomic variables that facilitate or hamper a person’s service use and might affect glycaemic control [18]. Four enabling factors were analysed: household income (in euros per month), educational level, employment status and marital status. Household income was ‘equivalised’ using the Organisation for Economic Co-operation and Development (OECD) square root scale to reflect differences in needs between households of different size [27]. Hence, the median value of the income class to which a given household belonged was divided by the square root of household size. Income classes ranged from <€750 to ≥€5000 per month, with each subsequent class representing a €250 income increase. Education was dichotomised as low/medium (elementary education, preparatory secondary vocational education, senior general secondary education or senior secondary vocational education) versus high (pre-university, higher professional or academic education) based on a participant’s highest completed type of education. With regard to employment status, two categories were distinguished: employed persons (self-employed/entrepreneurs, employees and civil servants) versus not employed persons (disabled, unemployed, rentiers, retirees, homemakers and others). Marital status could be either with partner (married or registered partners, or living together) or without partner (unmarried, widow(er), divorced, or other).

#### Health-related characteristics

The third category concerns health-related (or illness-level) factors, which–according to Anderson and Newman [18]–are the strongest predictors of health service use. Variables in this category were: diabetes duration (in years), diabetes-related complications, depression, HRQoL, and medication use, as well as multiple clinical measures determined by physical examination (i.e. weight, waist circumference, body mass index (BMI), and systolic and diastolic blood pressure) or laboratory assessment (i.e. HbA1c, total cholesterol, low-density lipoprotein (LDL) and high-density lipoprotein (HDL) cholesterol, and triglycerides).

Four diabetes-related complications were assessed–i.e. cardiovascular disease, neuropathic pain, retinopathy and chronic kidney disease–as described elsewhere [28,29]. Based on the Patient Health Questionnaire (PHQ) instrument for screening, diagnosing and measuring severity of depression, we categorised depression as: (1) no or minimal depressive symptoms (score 0–9); (2) minor depression (score 10–14); or (3) major depression (≥15) [30,31]. Besides the dichotomised HRQoL measures described earlier, a weighted overall HRQoL score was calculated from the EQ-5D-3L items, ranging from -0.33 to 1.00 on the basis of a Dutch validation study [15]. Medication use was categorised as none, oral and injectable (non-insulin) pharmacological agents (i.e. alfaglucosidase inhibitors, biguanides, dipeptidyl peptidase-4 (DPP4) inhibitors, glucagon-like peptide 1 analogues, and/or sulphonylurea derivatives), or insulin (with/without oral and injectable (non-insulin) pharmacological agents).

### Statistical analyses

Descriptive analyses were conducted to assess the biopsychosocial profile of diabetes patients by level of glycaemic control (HbA1c ≤7.0% [53 mmol/mol] vs >7.0% [53 mmol/mol]) in terms of the 38 included independent variables. Continuous variables are presented as means and standard deviations (SD); binary and categorical data as frequencies and valid percentages. Missing data were assumed to be missing at random and not imputed. Depending on the nature of the independent variables, different statistical tests were used to measure associations with glycaemic control. Thus, for continuous variables, independent samples t-tests were used; for binary and categorical variables, group comparisons were performed by chi-squared test and one-way ANOVA, respectively. A p-value <0.05 was set as level of significance. Analyses were conducted using IBM SPSS Statistics for Windows, version 23.0 (Armonk, NY).

LCA, also known as finite mixture modelling, was used to explore the existence of biopsychosocial profiles in the insufficient glycaemic control subgroup (HbA1c >7.0% [53 mmol/mol]), which differ in HRQoL. First, a one-class model was applied, after which the number of classes was sequentially increased up to a five-class model. To decide on the most parsimonious and best-fitting model, the Bayesian Information Criterion (BIC) was used for comparison across models, where the lowest value indicates the best fit [32]. The Lo-Mendell-Rubin likelihood ratio test (LMR-LRT) was also used to compare fit between neighbouring models. A significant p-value (p<0.05) indicates an improvement in fit for inclusion of one or more classes [32]. Entropy was used to determine the quality of classification. Higher entropy values indicate less ambiguity in class allocation [33]. LCA models were fitted using Mplus, version 7.3 [34]. Based on the results of the LCA, posterior probability of belonging to a given ‘latent class’ was determined for each patient and used as dependent variable in univariable logistic regression analyses to examine significant differences in biopsychosocial profile between HRQoL classes. Odds ratios (ORs) with 95% confidence intervals (CIs) were obtained using STATA version 14 [35].

## Results

Of The Maastricht Study participants with T2DM, 840 persons met the inclusion criteria. The study flowchart is included in Supplement 1 (S1 Fig). Mean age of the study population was 62.6 (±7.7) years. Males were overrepresented (68.6%). Mean HbA1c level was 7.1% (±3.2%) [54 (±12) mmol/mol]. Based on the Dutch diabetes care standard [2], 532 patients (63.3%) had sufficient glycaemic control (HbA1c≤7.0% [53 mmol/mol]), whereas 308 patients (36.7%) had insufficient control (HbA1c>7.0% [53 mmol/mol]).

### Biopsychosocial characteristics of diabetes patients by level of glycaemic control

Table 1 shows the distribution of person-related characteristics across subgroups. Patients with sufficient glycaemic control had a significantly higher level of self-efficacy compared to those with insufficient control (59.4±8.2 vs. 58.1±8.3; p = 0.047). There were no differences between subgroups in age, sex, smoking status, alcohol consumption, physical activity, mastery or social adequacy.

**Table 1:** Person-related patient characteristics by glycaemic control.

Characteristic	N	HbA1< = 7.0% [53 mmol/mol] (N = 532)	HbA1c >7.0% [53 mmol/mol] (N = 308)	Total (N = 840)	p-value
Age (years)	840	62.9±7.6	62.3±7.7	62.6±7.7	0.26
Sex	840				0.29
*Men*		*358 (67*.*3%)*	*218 (70*.*8%)*	*576 (68*.*6)*	
*Women*		*174 (32*.*7%)*	*90 (29*.*2%)*	*264 (31*.*4)*	
Smoking status	809				0.29
*Never*		*151 (29*.*5%)*	*73 (24*.*5%)*	*224 (27*.*7)*	
*Former*		*276 (54*.*0%)*	*172 (57*.*7%)*	*448 (55*.*4)*	
*Current*		*84 (16*.*4%)*	*53 (17*.*8%)*	*137 (16*.*9)*	
Alcohol consumption	809				0.27
*None*		*153 (29*.*9%)*	*100 (33*.*6%)*	*253 (31*.*3)*	
*Low*		*264 (51*.*7%)*	*155 (52*.*0%)*	*419 (51*.*8)*	
*High*		*94 (18*.*4%)*	*43 (14*.*4%)*	*137 (16*.*9)*	
Physical activity (hours/week)	672	12.1±7.7	11.8±8.0	12.0±7.8	0.57
Self-efficacy	672	59.4±8.2	58.1±8.3	58.9±8.2	0.047*
Mastery	680	25.6±4.8	25.2±5.0	25.5±4.9	0.27
Social adequacy	673	3.6±3.7	3.5±3.7	3.6±3.7	0.75

Continuous variables are presented as means and standard deviations (SD); binary and categorical data as frequencies and valid percentages.

*Significant at the P<0.05 level.

Table 2 shows the context-related characteristics of patients by HbA1c level. The sufficient glycaemic control subgroup had a significantly higher mean equivalent income (in euros) than the subgroup with insufficient control (1,899±906 vs. 1,736±763; p = 0.03). Moreover, there were significantly more high-educated persons and fewer low-educated persons among those with sufficient glycaemic control (p = 0.047). No subgroup differences were identified with regard to employment or marital status.

**Table 2:** Context-related patient characteristics by glycaemic control.

Characteristic	N	HbA1< = 7.0% [53 mmol/mol] (N = 532)	HbA1c >7.0% [53 mmol/mol] (N = 308)	Total (N = 840)	p-value
Equivalent income (euros)	551	1,899±906	1,736±763	1,841±861	0.03*
Educational level	809				0.047*
*Low/medium*		*373 (72*.*9)*	*235 (79*.*1)*	*608 (75*.*2)*	
*High*		*139 (27*.*1)*	*62 (20*.*9%)*	*201 (24*.*8)*	
Employment status	694				0.75
*Not employed*		*306 (68*.*6)*	*173 (69*.*8)*	*479 (69*.*0)*	
*Employed*		*140 (31*.*4)*	*75 (30*.*2)*	*215 (31*.*0)*	
Marital status	816				0.40
*No partner*		*109 (21*.*1)*	*71 (23*.*7)*	*180 (21*.*4)*	
*Partner*		*407 (78*.*9)*	*229 (76*.*3)*	*636 (77*.*9)*	

Continuous variables are presented as means and standard deviations (SD); binary and categorical data as frequencies and valid percentages.

*Significant at the P<0.05 level.

As to health-related characteristics (Tables 3–5), patients with insufficient glycaemic control had a significantly longer mean duration of diabetes (11.1±8.0 vs. 6.9±5.9 years; p<0.001), as well as a higher prevalence of cardiovascular disease (34.1 vs. 25.9%; p = 0.014), neuropathic pain (24.7 vs. 18.0%; p = 0.025), retinopathy (7.7 vs. 3.3%; p = 0.007) and chronic kidney disease (50.0 vs. 37.7%; p<0.001).

**Table 3:** Health-related patient characteristics by glycaemic control (continuous variables).

Characteristic	N	HbA1< = 7.0% [53 mmol/mol] (N = 532)	HbA1c >7.0% [53 mmol/mol] (N = 308)	Total (N = 840)	p-value
Diabetes duration	663	6.88±5.89	11.13±7.96	8.5±7.0	<0.001*
Diabetes-related distress (PAID)	710	9.3±11.6	15.3±15.2	11.6±13.4	<0.001*
EQ-5D-3L index score	791	0.86±0.20	0.83±0.19	0.85±0.20	0.05*
SF-36 Physical component score (total)	785	47.24±9.47	44.69±10.56	46.3±9.9	0.001*
SF-36 Mental component score (total)	785	53.11±8.79	51.55±9.44	52.5±9.0	0.02*
HbA1c (% [mmol/mol])	840	6.5±2.5 [47±4]	8.1±3.2 [65±12]	7.1±3.2 [54±12]	NA
Total cholesterol (mmol/l)	840	4.3±0.9	4.3±0.9	4.3±0.9	0.34
LDL cholesterol (mmol/l)	840	2.3±0.8	2.2±0.8	2.3±0.8	0.29
HDL cholesterol (mmol/l)	840	1.3±0.3	1.2±0.4	1.2±0.4	0.09
Triglycerides (mmol/l)	840	1.7±0.9	1.8±1.1	1.7±0.9	0.047*
Weight (kg)	838	87.1±15.2	91.6±17.7	88.7±16.3	<0.001*
Waist circumference (cm)	838	105.1±12.6	108.9±14.6	106.5±13.5	<0.001*
BMI (in kg/m^2^)	838	29.5±4.7	30.9±5.3	30.0±5.0	<0.001*
Systolic blood pressure (mmHg)	840	142.3±17.8	141.9±17.8	142.2±17.8	0.755
Diastolic blood pressure (mmHg)	840	77.1±9.5	76.3±9.5	76.8±9.5	0.265

Continuous variables are presented as means and standard deviations (SD).

*Significant at the P<0.05 level.

**Table 4:** Health-related patient characteristics by glycaemic control (binary variables).

Characteristic	N	Category	HbA1< = 7.0% [53 mmol/mol] (N = 532)	HbA1c >7.0% [53 mmol/mol] (N = 308)	Total (N = 840)	p-value
Cardiovascular disease	817	*No*	371 (74.1)	193 (65.9)	564 (71.0)	0.01*
		*Yes*	130 (25.9)	100 (34.1)	230 (29.0)	
Neuropathic pain	781	*No*	405 (82.0)	216 (75.3)	621 (79.5)	0.025*
		*Yes*	89 (18.0)	71 (24.7)	160 (20.5)	
Retinopathy	762	*No*	472 (96.7)	253 (92.3)	725 (95.1)	0.01*
		*Yes*	16 (3.3)	21 (7.7)	37 (4.9)	
Chronic kidney disease	816	*No*	325 (61.1)	147 (50.0)	472 (57.8)	0.001*
		*Yes*	197 (37.7)	147 (50.0)	344 (42.2)	
Diabetes-related distress (PAID)	710	*PAID score <40*	430 (97.3)	242 (90.3)	672 (94.6)	<0.001*
		*PAID score ≥40*	12 (2.7)	26 (9.7)	38 (5.4)	
EQ-5D-3L Mobility problems	796	*No*	356 (70.5)	186 (63.9)	542 (68.1)	0.055*
		*Yes*	149 (29.5)	105 (36.1)	254 (31.9)	
EQ-5D-3L Self-care problems	795	*No*	486 (96.4)	271 (93.1)	757 (95.2)	0.04*
		*Yes*	18 (3.6)	20 (6.9)	38 (4.8)	
EQ-5D-3L Usual activities problems	796	*No*	430 (85.3)	217 (74.3)	647 (81.3)	<0.001*
		*Yes*	74 (14.7)	75 (25.7)	149 (18.7)	
EQ-5D-3L Pain/discomfort	796	*No*	303 (60.1)	155 (53.1)	458 (57.5)	0.05*
		*Yes*	201 (39.9)	137 (46.9)	338 (42.5)	
EQ-5D-3L Anxiety/depression	796	*No*	430 (85.3)	229 (78.4)	659 (82.8)	0.01*
		*Yes*	74 (14.7)	63 (21.6)	137 (17.2)	
SF-36 Physical component score	*785*	*PCS≥50*	267 (53.6)	113 (39.4)	380 (48.4)	<0.001*
		*PCS<50*	231 (46.4)	174 (60.6)	405 (51.6)	
SF-36 Mental component score	785	*MCS≥42*	446 (89.6)	244 (85.0)	690 (87.9)	0.06
		*MCS<42*	52 (10.4)	43 (15.0)	95 (12.1)	

Binary variables are presented as frequencies and valid percentages.

*Significant at the P<0.05 level.

**Table 5:** Health-related patient characteristics by glycaemic control (categorical variables).

Characteristic	N		HbA1< = 7.0% [53 mmol/mol] (N = 532)	HbA1c >7.0% [53 mmol/mol] (N = 308)	Total (N = 840)	p-value
Depression	716	*No/minimal symptoms*	432 (93.3)	227 (89.7)	659 (92.0)	0.23
		*Minor depression*	19 (4.1)	15 (5.9)	34 (4.7)	
		*Major depression*	12 (2.6)	11 (4.3)	23 (3.2)	
Glucose-lowering medication	839	*None*	66 (12.4)	10 (3.2)	76 (9.1)	<0.001*
		*Oral and injectable (non-insulin)*	403 (75.9)	144 (46.8)	547 (65.2)	
		*Insulin*	62 (11.7)	154 (50.0)	216 (25.7)	

Categorical variables are presented as frequencies and valid percentages.

*Significant at the P<0.05 level.

HRQoL was reduced in the insufficient glycaemic control subgroup compared to patients with sufficient control. Thus, mean PAID scores indicated higher diabetes-related emotional distress (15.3±15.2 vs. 9.3±11.6; p<0.001) and there was a significantly higher percentage of patients at an emotional burn-out level, as indicated by a PAID score ≥40 (9.7 vs. 2.7%; p<0.001). Moreover, mean summary scores on all domains of HRQoL measured by the EQ-5D-3L and SF-36 were significantly lower among patients with insufficient glycaemic control, as was the overall EQ-5D-3L index score.

Medication use was different between subgroups (p<0.001): in particular, the percentage of patients on insulin was greater in patients with insufficient glycaemic control compared to those with sufficient control (50.0 vs. 11.7%). In terms of clinical measures, patients with insufficient glycaemic control differed significantly from their counterparts in terms of weight (91.6±17.7 vs. 87.1±15.2; p<0.001), waist circumference (108.9±14.6 vs. 105.1±12.6; p<0.001), BMI (30.9±5.3 vs. 29.5±4.7; p<0.001) and triglycerides (1.8±1.1 vs. 1.7±0.9; p = 0.047).

### HRQoL in patients with insufficient glycaemic control: Biopsychosocial profiles

Among patients with insufficient glycaemic control (HbA1c >7.0% [53 mmol/mol]; N = 308), LCA was used to explore the existence of distinct biopsychosocial profiles, which differ in terms of HRQoL. LCA models were run with one to five classes. The model fit indices showed that the two- and three-class models had the best fit (S1 Table). The two-class model was chosen for further analysis, because of little distinction in patterns and item probabilities between class 2 and class 3, as well as the small percentage of patients in class 3 based on most likely class membership (4.9%).

**Fig 1:**
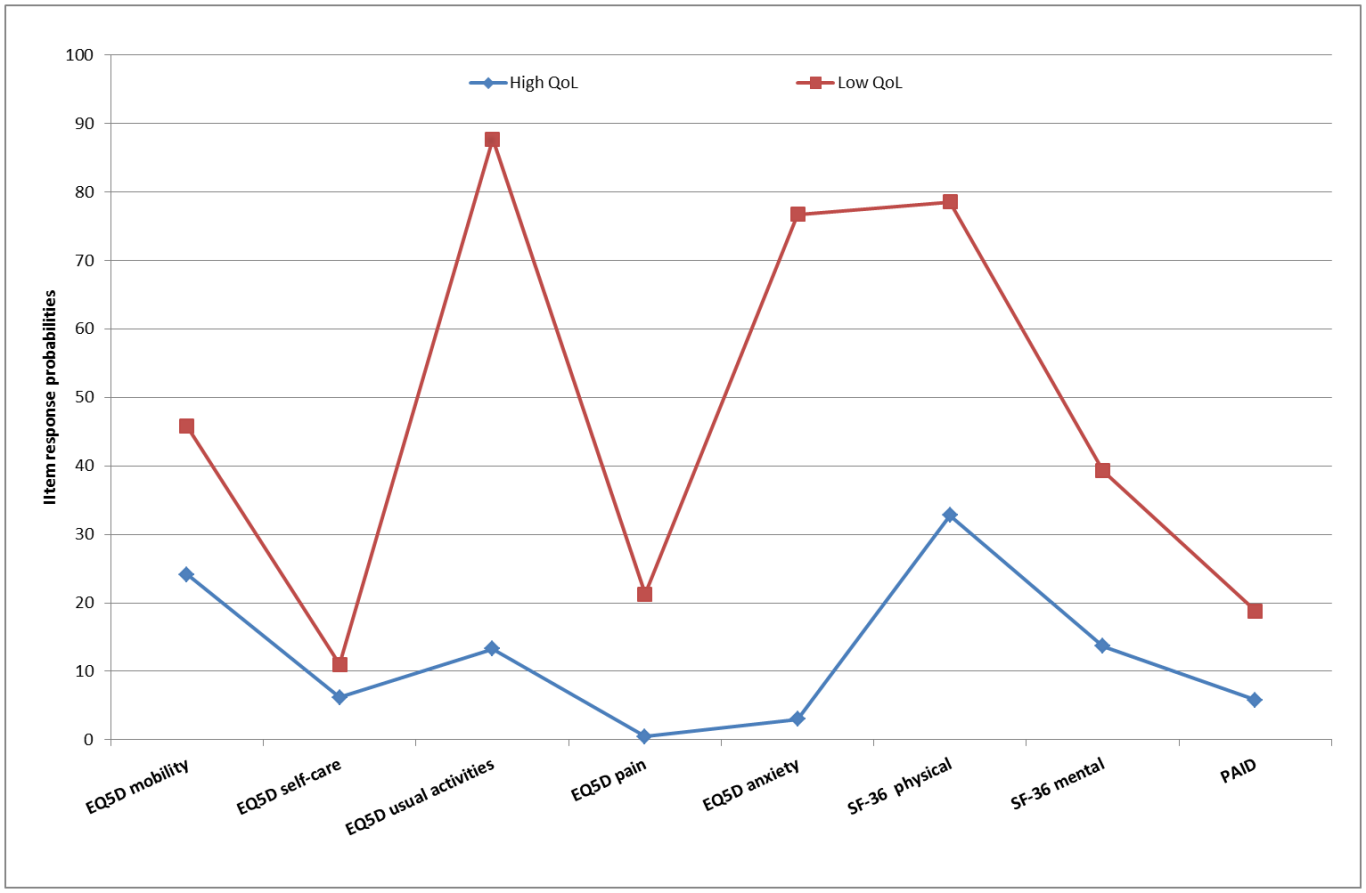
Two-class model for HRQoL in patients with insufficient glycaemic control (HbA1c>7.0% [53 mmol/mol]). High HRQoL class, N = 220 (71.4%); low HRQoL class, N = 88 (28.6%).

Fig 1 shows the item response probability plot for the final two-class model. Values on the y-axes represent the likelihood, by class, of patients experiencing problems related to included HRQoL domains. Two distinct classes were identified: patients with ‘low’ HRQoL (28.6%; N = 88) versus patients with ‘high’ HRQoL (71.4%; N = 220). Classes differed most in the probability of experiencing problems with usual activities, anxiety and physical functioning, which was greater for patients with low HRQoL (~70–90%; Fig 1). On the other hand, the chance of problems with self-care and pain, as well as for severe diabetes-related distress (PAID score ≥40), was relatively low and comparable in both classes, although consistently greater in the low HRQoL class. The likelihood of mobility issues was around 50% in the low HRQoL class versus circa 25% in the high HRQoL class.

Tables 6–8 summarize the biopsychosocial characteristics of the identified HRQoL classes and show which characteristics were associated with HRQoL-based class membership (high HRQoL class is used as reference category). With regard to person-related characteristics, women had higher odds than men to be in the low HRQoL class (OR 2.32; 95% CI 1.36–3.94; p = 0.002), as did current smokers compared to non-smokers (OR 2.24; 95% CI 1.03–4.88; p = 0.04). Other person-related factors associated with greater odds of being in the low HRQoL class were no versus low or high alcohol consumption, less than 7 hours of physical activity per week versus 14 hours or more, and lower mastery, self-efficacy and social adequacy (Table 6).

**Table 6:** Person-related characteristics of T2DM patient across different classes of HRQoL.

	N	Category	Biopsychosocial characteristics		OR (95% CI)	p-value
			*High HRQoL class* *(N = 220)*	*Low HRQoL class* *(N = 88)*	*Low HRQoL class*	
Age (years)	308	*41–49*	20 (9.1)	3 (3.4)	Reference	
		*50–64*	108 (49.1)	41 (46.6)	2.50 [0.70–8.91]	0.16
		*65–76*	92 (41.8)	44 (50.0)	3.11 [0.87–11.07]	0.08
Sex	308	*Male*	167 (75.9)	51 (58.0)	Reference	
		*Female*	53 (24.1)	37 (42.0)	2.32 [1.36–3.94]	0.002*
Smoking status	298	*Never*	56 (26.4)	17 (19.8)	Reference	
		*Former*	124 (58.5)	48 (55.8)	1.25 [0.66–2.37]	0.50
		*Current*	32 (15.1)	21 (24.4)	2.24 [1.03–4.88]	0.04*
Alcohol consumption	298	*None*	58 (27.4)	42 (48.9)	Reference	
		*Low*	120 (56.6)	35 (40.7)	0.40 [0.23–0.96]	0.001*
		*High*	34 (16.0)	9 (10.5)	0.31 [0.13–0.74]	0.008*
Physical exercise	240	*<7 h/w*	48 (27.6)	28 (42.4)	Reference	
		*7–13 h/w*	66 (37.9)	20 (30.3)	0.53 [0.27–1.05]	0.07
		*≥14 h/w*	60 (34.5)	18 (27.3)	0.48 [0.23–0.98]	0.04*
Mastery	242		174±26.3	68±22.4	0.48 [0.36–0.65]	<0.001*
Self-efficacy	238		59.6±7.8	54.3±8.6	0.92 [0.88–0.96]	<0.001*
Social inadequacy	240		172±3.1	68±4.5	1.11 [1.03–1.20]	0.006*

Continuous variables are presented as means and standard deviations (SD); binary and categorical data as frequencies and valid percentages.

*Significant at the P<0.05 level.

**Table 7:** Context-related characteristics of T2DM patient across different classes of HRQoL.

	N	Biopsychosocial characteristics		OR (95% CI)	p-value
		*High HRQoL class* *(N = 220)*	*Low HRQoL class* *(N = 88)*	*Low HRQoL class*	
Equivalent income	195	1837±791	1499±640	0.10 [0.10–0.10]	0.007*
Educational level (*Low/medium*)	297	159 (75.7)	76 (87.4)	2.28 [1.12–4.67]	0.02*
Employment status (*Unemployed*)	248	109 (61.2)	64 (91.4)	8.05 [3.23–20.10]	<0.001*
Marital status (*No partner*)	300	46 (21.6)	25 (28.7)	1.42 [0.80–2.51]	0.23

Continuous variables are presented as means and standard deviations (SD); binary and categorical data as frequencies and valid percentages.

*Significant at the P<0.05 level.

**Table 8:** Health-related characteristics of T2DM patient across different classes of HRQoL.

	N	Category	Biopsychosocial characteristics		OR (95% CI)	p-value
			*High HRQoL class (N = 220)*	*Low HRQoL class (N = 88)*	*Low HRQoL class*	
Diabetes duration	251	*<5 years*	52 (28.9)	11 (15.5)	Reference	
		*5–9 years*	42 (23.3)	18 (25.4)	2.04 [0.86–4.84]	0.11
		*≥ 10 years*	86 (47.8)	42 (59.2)	2.41 [1.13–5.13]	0.02*
Cardiovascular disease	293		61 (29.5)	39 (45.3)	2.08 [1.23–3.52]	0.006*
Neuropathic pain	287		36 (17.8)	35 (41.2)	3.26 [1.85–5.76]	<0.001*
Retinopathy	274		12 (6.1)	9 (11.7)	2.09 [0.83–5.24]	0.12
Chronic kidney disease	294		93 (44.3)	54 (64.3)	2.48 [1.46–4.21]	0.001*
Depression	253	*No/minimal*	170 (94.4)	57 (78.1)	Reference	
		*Minor depression*	6 (3.3)	9 (12.3)	4.31 [1.44–12.87]	0.009*
		*Major depression*	4 (2.2)	7 (9.6)	6.21 [1.74–22.18]	0.005*
Glucose-lowering medication (*Insulin*)	308		100 (54.0)	54 (61.3)	1.98 [1.19–3.30]	0.009*
HbA1c (% [mmol/mol])	308		8.0±3.1 [64±10]	8.4±3.5 [68±15]	1.03 [1.01–1.05]	0.009*
Total cholesterol (mmol/l)	308		4.2±0.9	4.3±0.9	1.07 [0.82–1.39]	0.62
LDL cholesterol (mmol/l)	308		2.3±0.8	2.2±0.8	0.94 [0.68–1.30]	0.70
HDL cholesterol (mmol/l)	308		1.2±0.4	1.2±0.4	0.97 [0.51–1.83]	0.93
Triglycerides (mmol/l)	308		1.8±1.1	2.0±1.1	1.21 [0.96–1.52]	0.11
Weight (kg)	301		90.2±16.7	94.4±17.9	1.02 [1.00–1.03]	0.04*
Waist circumference (cm)	303		107. 2±13.5	113.8±16.3	1.03 [1.01–1.05]	0.001*
BMI (kg/m^2^)	308		30.1±4.7	33.0±6.2	1.12 [1.06–1.18]	<0.001*
Systolic blood pressure (mmHg)	308		142.8±17.9	139.8±17.5	0.99 [0.98–1.01]	0.23
Diastolic blood pressure (mmHg)	308		76.8±9.3	75.3±9.8	0.98 [0.95–1.01]	0.17

Continuous variables are presented as means and standard deviations (SD); binary and categorical data as frequencies and valid percentages.

*Significant at the P<0.05 level.

Apart from marital status, all context-related characteristics (Table 7) were significantly different between HRQoL classes. Lower equivalent income was associated with higher odds of being in the low HRQoL class (OR 0.10; 95% CI 0.10–0.10; p = 0.007), as was a low or medium educational level (OR 2.28; 95% CI 1.12–4.67; p = 0.02) and unemployment (OR 8.05; 95% CI 3.23–20.10; p<0.001).

As for health-related characteristics (Table 8), a diabetes duration of ≥10 years relative to <5 years was associated with higher odds for the low HRQoL class (OR 2.41; 95% CI 1.13–5.13; p = 0.02). Patients with cardiovascular disease, neuropathic pain or chronic kidney disease also had significantly higher odds to be in the low HRQoL class, as did patients with minor or major depression (ORs ranging from 2.08 to 6.21). Medication-wise, use of insulin instead of no or other diabetes medication was associated with higher odds for the low HRQoL class (OR 1.98; 95% CI 1.19–3.30; p = 0.009). Of the clinical measures, higher HbA1c, BMI, weight or waist circumference was associated with greater odds of belonging to the low HRQoL class (ORs from 1.02 to 1.12).

## Discussion

Findings from this study suggest that significant differences exist in biopsychosocial characteristics between subgroups of diabetes patients by level of glycaemic control. Most characteristics were health-related, including HRQoL, complications, medication, and BMI. Of the assessed person- and context-related characteristics, self-efficacy respectively income and education level differed between glycaemic control subgroups, albeit modestly. Identified associations were consistently negative: a worse status on any of the significant variables was associated with less glycaemic control. Zooming in further on the insufficient glycaemic control subgroup, we identified two distinct patient classes in terms of HRQoL: one with a low probability of HRQoL problems and one with a higher probability of such problems. A broad range of biopsychosocial factors was associated with low HRQoL class membership, including lower levels of mastery, self-efficacy and social adequacy, lower income and education levels, longer disease duration, presence of various complications, and insulin use.

In 2012, the European Association for the Study of Diabetes (EASD) and American Diabetes Association (ADA) published a position statement on hyperglycaemia management in T2DM, which described the need to individualise treatment targets and strategies [8]. Yet in most countries, diabetes management remains highly standardised and does not comprehensively account for heterogeneity within the diabetes population [36,37]. Our findings support the need for more individualised management, by showing that patients with insufficient glycaemic control differ considerably from those with sufficient control. Differences exist not only in health-related variables, as emphasised by the EASD and ADA, but also on a psychosocial and socioeconomic level. Particularly lower self-efficacy, income and/or education levels seem to be associated with less glycaemic control. This is supported by previous research demonstrating the effects of self-efficacy on diabetes self-management and, consequently, glycaemic control [38]. Increasing evidence supports the notion that people’s control beliefs are a fundamental mechanism underlying socioeconomic differences in health [39–41]. This might be particularly true for T2DM patients, as recent work suggests that among chronically ill, control beliefs are even more important determinants of HRQoL than social support or income [42].

To our knowledge, this is the first LCA among T2DM patients with insufficient glycaemic control. Findings suggest that in terms of HRQoL–described as an outcome that ‘actually matters to patients’ [43]–distinct classes exist within this subgroup: about a quarter of patients has serious problems in multiple HRQoL domains, whereas the others do not (yet) experience any limitations. This finding might partly explain why previous studies into the relation of glycaemic control with HRQoL, which did not account for ‘latent subclasses’, have found weak and inconsistent associations [44,45]. Looking at the specific domains in which problems were most likely to occur, i.e. with usual activities, anxiety and physical functioning, diabetes-related complications might be important predictors of low HRQoL. Indeed, previous research suggests that complications are more strongly associated with HRQoL than HbA1c, and that even minor complications can have a significant impact on HRQoL [46,47]. Given their higher complication rates and longer disease duration, it is not surprising that patients with insufficient glycaemic control–particularly those with low HRQoL–were more likely to use insulin. However, the overrepresentation of insulin users in this class might also suggest that insulin is an inadequate ‘last resort’ for some patients.

Patients with low versus high HRQoL in the insufficient glycaemic control subgroup also differed in person- and context-related characteristics–more profoundly even than when comparing patients by level of glycaemic control. Here again, control beliefs might mediate socioeconomic health differences. Living with diabetes poses many challenges for patients in areas like nutrition, glycaemic monitoring and medication adherence, which tend to become increasingly difficult and burdensome as glycaemic control deteriorates [48]. However, the knowledge, skills, confidence and means–both financially and socially–needed to adequately respond to these challenges are not distributed equally among the population, which might contribute to differences in HRQoL among those with insufficient glycaemic control. Indeed, estimates from the United Kingdom show that morbidity from diabetes-related complications is more than three times higher among the less well-off compared to the wealthiest [49].

This study has a number of strengths and limitations. We drew on the comprehensive phenotyping approach of The Maastricht Study [11] and used a relatively large sample size, allowing for the investigation of multiple subgroups and classes. Although there is no formal benchmark for adequate sample size in LCA, Finch and Bronk [50] concluded–based on a number of simulation studies–that 500 participants is ‘a worthy goal in practice’. In terms of methods, LCA is a sophisticated analytic technique, which allowed us to improve understanding of previously unobserved subgroups in the diabetes population. An important advantage of LCA over traditional types of cluster analysis is its probability-based classification, which better captures uncertainty [51]. Given the complex and difficult to differentiate interactions that might exist between many of the included variables, investigating causal relations via multivariable analysis was beyond the scope of this explorative study. On one hand, this is a limitation of the study, as it precludes any conclusions about which patient characteristics are the strongest predictors of insufficient glycaemic control and/or low HRQoL, and which are confounders. On the other hand, our univariable exploration of a broad range of possibly relevant characteristics provides a sound basis for more targeted, hypothesis-driven future investigations of causal relations using multivariable models, and is in line with the biopsychosocial paradigm that is gaining increasing traction in health care [52]. Univariable analyses also enabled us to maintain a relatively large overall sample size, despite missing values in some independent variables. A final limitation relates to the relative underrepresentation of people with severe diabetic complications in The Maastricht Study. As a result, the study sample may be healthier than the average diabetes population, which could mean that some of the associations measured between patient factors and health outcomes are underestimations.

In conclusion, this explorative study shows that insufficient glycaemic control, particularly in combination with low HRQoL, is associated with a generally less positive biopsychosocial profile. Further studies, especially multivariable analyses, are needed to better understand the complex and multidimensional causal pathways between relevant biopsychosocial characteristics of T2DM patients and their health outcomes. Perhaps even more importantly, we need to learn more about the self-perceived care needs and preferences of different patient subgroups, and how we can meet them with well-aligned care and support strategies. With regard to the latter, a large-scale study is currently being conducted in the Netherlands (‘PROFILe’), which builds on the findings of the present study to develop an instrument supporting more tailored, person-centred chronic care [53] The first results of PROFILe are expected in 2017.

## Supporting information

S1 Fig Study flowchart. (DOCX)

S1 Table Statistical criteria for latent class models with 1 to 5 latent classes. ^a^Bayesian Information Criterion; ^b^Lo-Mendell-Rubin Likelihood Ratio Test; *Significant at the P<0.05 level.
(DOCX)
