# Supplementary material for: Differences in biopsychosocial profiles of diabetes patients by level of glycaemic control and health-related quality of life: The Maastricht Study
Source: PLoS One. 2017 Jul 27;12(7):e0182053. doi: 10.1371/journal.pone.0182053 (PMC5531491; doi:10.1371/journal.pone.0182053)
Supplement: S1 Fig — (DOCX) [file pone.0182053.s001.docx]

# S1 Figure

Participants with T2DM

in the Maastricht Study

N=975

Exclusion criteria:

*No previous diagnosis of T2DM (N=133)*

*No HbA1c measurement (N=2)*

Study population

N=840
