# Supplementary material for: Differences in biopsychosocial profiles of diabetes patients by level of glycaemic control and health-related quality of life: The Maastricht Study
Source: PLoS One. 2017 Jul 27;12(7):e0182053. doi: 10.1371/journal.pone.0182053 (PMC5531491; doi:10.1371/journal.pone.0182053)
Supplement: S1 Table — aBayesian Information Criterion; bLo-Mendell-Rubin Likelihood Ratio Test; *Significant at the P<0.05 level. (DOCX) [file pone.0182053.s002.docx]

# S1 Table

| **Number of**  **Classes** | **BIC^a^** | **LMR-LRT^b^** | **Entropy** | **Percentage patients per class based on most likely class membership** |
| --- | --- | --- | --- | --- |
| **1** | 2.292.709 | NA | NA | 100 |
| **2** | 2140.802 | -1123.434* | 0.757 | 71.4-28.6 |
| **3** | 2164.218 | -1021.695* | 0.803 | 67.5-27.6-4.9 |
| **4** | 2196.614 | -1007.618 | 0.793 | 47.4-29.2-18.5-4.9 |
| **5** | 2235.191 | -998.030 | 0.821 | 46.7-29.2-14.0-6.5-3.6 |
